# Quality of life in patients with fibromyalgia: Contributions of disease symptoms, lifestyle and multi-medication

**DOI:** 10.3389/fpsyg.2022.924405

**Published:** 2022-10-03

**Authors:** Fátima Fernandez-Feijoo, Noelia Samartin-Veiga, María Teresa Carrillo-de-la-Peña

**Affiliations:** Brain and Pain Lab, Department of Clinical Psychology and Psychobiology, University of Santiago de Compostela, Santiago de Compostela, Spain

**Keywords:** chronic pain, quality of life, lifestyle, anxiety, depression, multi-medication

## Abstract

Fibromyalgia (FM) is a disease characterized by the presence of chronic and widespread musculoskeletal pain, which causes a high negative impact on the quality of life (QoL). Although there are many studies about the QoL of patients with FM, it is unknown which variables have a main influence on it. Therefore, in the present study, we aimed to determine which FM symptoms predict a worse QoL and also to establish whether lifestyle and multi-medication are associated to QoL. We assessed a sample of 134 women with FM using a semi-structured clinical interview to explore lifestyle (diet, exercise, smoking) and medication use, and questionnaires to cover the main symptoms of this disease and QoL (SF-36). We found that the patients with FM had a poor QoL, being “physical pain” and “vitality” the most affected domains. A linear regression analysis showed that depression and anxiety assessed by HADS were the FM symptoms which most significantly predicted QoL, explaining 49% of the variance. Concerning lifestyle/medication influences, we found that multiple drug treatment and smoking also predicted a worse QoL (14%). Moreover, patients who practiced exercise regularly showed better QoL than patients who did not (regardless of the severity of FM). Thus, our results suggest that treatment strategies to improve QoL in FM should be focused on improving psychological distress, promoting regular exercise and reducing smoking and multi-medication. The data highlights the role of positive self-management practices to improve QoL in FM.

## Introduction

Fibromyalgia (FM) is a disease with a prevalence between 2 and 4% in the general population (Wolfe et al., 1995; Galvez-Sánchez et al., 2020), present mainly in women (about 3–8% for women and 0.5–5% for men in United States) (Kueny et al., 2021) and characterized by the presence of chronic and widespread musculoskeletal pain, sleep disturbances, constant fatigue, cognitive alterations, and mood disorders (Wolfe et al., 2011, 2016). This condition causes a high negative impact on the quality of life (QoL) of people who suffer from it (Tander et al., 2008), so understanding the factors influencing QoL may improve management of the patients with this chronic condition.

Much research has been done to understand which factors are associated with the QoL of people with FM. Widespread pain has a main negative impact on QoL, since it affects activities of daily living and social interactions, and increases stress and anxiety (Clauw et al., 2019; Sempere-Rubio et al., 2019). Depression and anxiety can aggravate the primary symptoms of the disease (pain, fatigue and insomnia) and, in turn, reduce QoL (Galvez-Sánchez et al., 2020). A previous study using the Hospital Anxiety and Depression Scale (HADS) showed that anxiety and/or depression are good predictors of several subscales of the SF-36 (such as general health, vitality, social functioning, emotional role functioning and mental health) and of the Fibromyalgia Impact Questionnaire (FIQ) (Campos and Vázquez, 2012). Other symptoms, such as sleep problems, also show a close relationship with poor QoL (Wagner et al., 2012; D’Aoust et al., 2017). It has been suggested that sleep difficulties lead to increased fatigue and anxiety (Turkyilmaz et al., 2012) and pain ratings (Keskindag and Karaaziz, 2017), thus decreasing QoL. However, although there is evidence of a segregated effect of the above symptoms on QoL, we are not aware of previous research that examined which of them has the greatest weight to predict QoL in patients with FM.

In addition, lifestyle has emerged as a crucial predictor of QoL in FM. In one hand, there is evidence of a link between chronic pain and diet (Logan, 2003; VanDenKerkhof et al., 2011; Rossi et al., 2015). In this vein, the body mass index (BMI) has been suggested as a potential explanatory factor in the relationship between diet and chronic pain (VanDenKerkhof et al., 2011). In fact, the review by Rossi et al. (2015) supports that obesity could be related to the severity of FM, worsening the QoL of patients suffering from it. Regular physical exercise also has a significant impact in QoL in people with FM (Galvez-Sánchez et al., 2019; Sempere-Rubio et al., 2019). Women with FM are less physically active than healthy women (McLoughlin et al., 2011), and have lower perceived functional capacity and physical performance (Jones et al., 2010). Furthermore, those patients who are physically active appear to modulate pain more adequately than those who are more sedentary (McLoughlin et al., 2011). In this vein, physical activity in FM was positively related to brain responses generated during pain distraction, whereas sustained sedentary behavior was negatively associated with pain regulation (Ellingson et al., 2012). In the other hand, in two recent studies (Ge et al., 2019; Croghan et al., 2021) that surveyed large samples of patients with FM (668 and 1068, respectively), tobacco use was also associated with greater pain and other symptom severity, and with worse QoL.

Moreover, to our knowledge the role of polypharmacy on QoL has not been explored so far. Combined pharmacological treatment is a common therapeutic strategy in FM. In other chronic pain diseases, increased consumption of medicines is associated to multiple negative consequences on patients’ health (increased risk of side effects and harms), and not always increases QoL (Chaparro et al., 2012; Giummarra et al., 2015; Ersoy and Engin, 2018). Again, although the above lifestyle/medication patterns may have a role in QoL in FM, the relative contribution of them is unknown.

Therefore, the main objectives of this study were: (1) to determine which of the symptoms of FM (pain, sleep dysfunction, anxiety, and depression) are associated to a worse QoL, assessed with the SF-36 questionnaire; (2) to analyze whether having a balanced diet, making exercise regularly, and not smoking contribute to better QoL; (3) to determine the role of multi-medication on the QoL of patients with FM. We expected to find a positive association of QoL with a healthier lifestyle and reduced medication use.

## Materials and methods

### Participants

We recruited a sample of 134 women. The inclusion criteria used in the study were diagnosis of FM (normally made by the general practitioner and confirmed by a rheumatologist), and compliance with ACR criteria for FM established in 1990 and 2010 (Wolfe et al., 1990, 2010); age between 25 to 65 years; and a stable medication pattern for at least 2 months. The exclusion criteria were the presence of immune pathology or comorbidities that could explain the main symptomatology of FM; the presence of brain damage, dementia, or neurological disease; and the presence of psychiatric disorders (other than anxiety and depression).

The data used in this study correspond to the pre-treatment evaluation of a clinical trial to assess the effect of transcranial electrical stimulation in patients with FM (pre-registered at http://www.encepp.eu/, with code 24294). The study was approved by the Galician Research Ethics Committee with code 2014/488, according to the Declaration of Helsinki.

### Procedure and materials

Participants were contacted by phone and scheduled, if they agreed to participate, for an evaluation session. First, they were provided with information about the study and signed the informed consent form. Initially, an extensive clinical interview was used to assess the core symptoms of FM and the presence of comorbidities, as well as medication use. In addition, we included questions about corporal parameters (height and weight) to calculate the BMI; and also, lifestyle-related items, such as a balanced diet (the answer options were yes or not), regular exercise (the answer options were yes or not, which one/s, and how often), and tobacco use (smoking or not, time smoking, number of cigarettes). Concerning medication use, multi-medication was considered if the patients’ medication pattern consisted of more than three different types of drugs (classified as analgesics, anxiolytics, antidepressants, antipsychotics, sedatives, and others).

The following self-reported scales were also administered (in their Spanish validated versions):

The SF-36 Health Questionnaire (Ware and Sherbourne, 1992; López-García et al., 2003) encompasses the most relevant health concepts included in the Medical Outcomes Study (MOS). It is composed of 8 scales (which contain between 2 and 10 items), related to physical function, physical role, body pain, general health, vitality, social function, emotional role, mental health, and a scale (made up of a single item) to compare current health with a year ago health. The score of each subscale ranges from 0 to 100, where 0 represents the worst possible level of QoL and 100, the best. In this study we considered each scale separately and also an average of the 8 scales to have a global score in QoL.

A Numeric Rating Scale (NRS) created *ad hoc* was used to assess the clinical pain status of the participants. They were asked to indicate their pain intensity in the previous month being 0 no pain and 10, the most intense pain.

The Pittsburgh Sleep Quality Index (PSQI) (Buysse et al., 1989; Macías and Royuela, 1996) was used to assess sleep quality and dysfunction in the past month. It is composed of 7 subscales (from 0 - no difficulty- to 3 - maximum difficulty-), which describe different aspects of sleep problems. The overall score ranges from 0 to 21, with higher scores indicating poorer sleep quality.

The Hospital Anxiety and Depression Scale (HADS) (Zigmond and Snaith, 1983; Herrero et al., 2003) is made up of 14 items and allows the detection of anxiety disorders and depression in primary care settings. HADS is a Likert-type scale ranging from 0 to 3, where patients have to describe the feelings experienced during the last week. Its score ranges from 0 to 42, with higher scores indicating a worse emotional state.

The Fibromyalgia Survey Questionnaire (FSQ) (Wolfe et al., 2011; Carrillo-de-la-Peña et al., 2015) is a self-reported questionnaire composed by two scales: WPI (Widespread Pain Index) and SSS (Symptom Severity Scale). The WPI indicates the number of body areas that present pain; and its score ranges between 0 (lack of painful areas) and 19 (all areas are painful). The SSS scale includes the level of tiredness/fatigue, concentration, attention or memory problems, and un-refreshing sleep, in addition to other symptoms such as abdominal pain, depression and headache; and its score ranges between 0 (no presence of these symptoms) and 12. We used the sum of WPI and SSS as an index of severity of the disease.

### Data analysis

First, we performed descriptive statistics of the SF-36 (sub-scales and overall mean score) and of all the variables under study (means and standard deviations for continuous variables; frequencies for binary variables). As a response to objective 1, we performed a linear regression analysis (forward) to determine which symptom (pain assessed by NRS, sleep quality assessed by PSQI, mood assessed by HADS) best predict the dependent variable (quality of life assessed by SF-36). Second, a logistic regression analysis using binary variables related to lifestyle (diet, exercise, smoking) and multi-medication was done to predict low vs. high QoL (dichotomized by the SF-36 median). Finally, we established subgroups of patients with FM according to lifestyle/medication variables, and calculated student’s *t*-test for independent samples to find out the differences between groups in QoL (assessed by SF-36 subscales and overall mean score) and FM severity (assessed by FSQ).

For all analyses we used the statistical package SPSS Statistics (v. 22), and the significance level was established as *p* value < 0.05.

## Results

The mean age of the participants was 50.38 years (SD = 8.68). As may be seen in Table 1, all the SF-36 subscales, with the exception of “emotional role” had an average score below 50 (half of the maximum score), being “body pain” and “vitality” the subscales with the lowest scores. The SF-36 mean score was 35.29. The participants showed moderate-high severity of FM, with a mean FSQ score of 21.01.

**TABLE 1:** Descriptive statistics of questionnaires of quality of life (QoL) (SF-36 subscales and global mean score), and Fibromyalgia severity (FSQ), as well as of the different variables considered as factors of influence on QoL.

QoL/FM severity	Mean (SD)	Variables affecting QoL	Mean (SD)
SF-36 (*n* = 129)		FM symptoms	
Physical Role	28.34 (20.36)	Pain (NRS) (*n* = 126)	7.29 (1.88)
Physical Function	42.36 (19.92)	Anxiety and depression (HADS) (*n* = 130)	21.22 (7.28)
Body Pain	22.36 (15.92)	Sleep quality (PSQI) (*n* = 127)	13.09 (4.37)
		
General Health	29.82 (16.71)	**Lifestyle**	**Percentage** **(yes-no)**
		
Vitality	25.27 (13.55)	Balanced Diet (*n* = 129)	74.4%–25.6%
Social Function	39.92 (24.01)	Regular Exercise (*n* = 131)	69.5%–30.5%
Mental Health	40.09 (14.87)	Overweight (*n* = 130)	58.5%–41.5%
Emotional Role	54.26 (28.30)	Smoke (*n* = 129)	22.5%–77.5%
Mean Score	35.29 (13.62)	Multi-medication (*n* = 131)	38.2%–61.8%
**FSQ** (*n* = 131)	21.01 (4.96)		

BMI, Body Mass Index; FSQ, Fibromyalgia Survey Questionnaire; NRS, Numeric Rating Scale; PSQI, Pittsburgh Sleep Quality Index; SD, standard deviation; HADS, hospital anxiety and depression scale.

Concerning lifestyle, most of the patients declared having a balance diet (74.4%), and making exercise regularly (69.5%), while smoking (22.5%) and polypharmacy (38.2%) were less frequent. The mean body mass index (BMI) was 25.98, and 58.5% of the participants were overweight (i.e., a BMI equal to or greater than 25).

The multiple linear regression analysis, using NRS-pain, PSQI, and HADS as independent variables and SF-36 mean score as the dependent variable, showed that HADS entered first in the model, explaining 49% of the variance. Pain and sleep quality were, respectively, the second and third variables included in the model. Altogether, the 3 variables explained 68% of the variance (see Table 2).

**TABLE 2:** Linear regression analysis (Dependent variable: SF-36; Predictors: Pain NRS, PSQI score, HADS score). Model summary and coefficients of the significant variables.

	Symptoms variables models (*n* = 120)									
	F	R^2^	Δ R^2^	B	Standard error	β	t	P	*1- β*	*f ^2^*
**Model 1**	113.220 (1,119)	0.490	0.485						1	0.960
HADS score				–1.323	0.124	–0.700	–10.640	0.000		
**Model 2**	98.636 (2,119)	0.628	0.621						1	1.638
HADS score				–0.992	0.118	–0.525	–8.420	0.000		
Pain NRS				–3.033	0.460	–0.411	–6.587	0.000		
**Model 3**	82.192 (3,119)	0.680	0.672						1	2.125
HADS score				–0.695	0.129	–0.368	–5.379	0.000		
Pain NRS				–2.482	0.447	–0.336	–5.556	0.000		
PSQI score				–0.968	0.222	–0.304	–4357	0.000		

HADS, Hospital Anxiety and Depression Scale; NRS, Numeric Rating Scale; PSQI, Pittsburgh Sleep Quality Index. R^2^, model adjustment; ΔR^2^, corrected model adjustment; B, degree of change in the outcome variable for every 1-unit of change in the predictor variable; β, standardized B coefficient; f^2^, effect size parameter (f^2^ small < 0.02; f^2^ medium > 0.15 < 0.35; f^2^ large > 0.35).

To clarify the impact of lifestyle/medication on QoL, we performed logistic binary regression analysis on groups stablished by the median value of SF-36 (low vs. high QoL). As may be seen in Table 3, only multi-medication and smoking were significant predictors of QoL, although the variance explained by the two variables was very small (14%).

**TABLE 3:** Logistic binary regression analysis (Dependent variable: SF-36 dichotomic; Predictors: balanced diet, regular exercise, overweight, smoke, multi-medication).

	Lifestyle variables models (*n* = 122)				
	R^2^ cox and snell	R^2^ nagelkerke	χ^2^ (p)	OR (95% CI)	P
**Model 1**	0.067	0.089	8.424 (0.004)		
Multi-medication				0.330 (0.153–0.711)	0.005
**Model 2**	0.105	0.140	13.480 (0.001)		
Multi-medication				0.340 (0.156–0.744)	0.007
Smoking				0.352 (0.137–0.902)	0.030

Model summary of the significant variables. OR, odd ratios; CI, confidence interval.

The comparisons of subgroups according to lifestyle/medication patterns (Table 4) showed that participants keeping or not a balanced diet did not show differences in QoL nor in disease severity. On the contrary, student’s *t*-test for independent samples showed that those participants who exercised regularly presented a significantly higher score on the SF-36 mean than those who did not exercise regularly (*M* = 36.93 vs. 30.60; *p* = 0.014), even if they did not differ in the severity or impact of the disease. Also, patients doing exercise regularly presented better scores in “physical function,” “physical role,” “body pain,” “vitality,” and “social function.” Patients with FM who smoke also showed poorer overall QoL (*M* = 29.56 vs. 36.79; *p* = 0.011), scoring worst on “social function”, but they did not differ in the severity of the syndrome (assessed by the FSQ). Finally, patients who consumed three or more different type of drugs showed worse QoL (*M* = 30.64 vs. 37.82; *p* = 0.003), scored worst on “physical function,” “social function,” “mental health” and “emotional role”; but they did not differ in the severity of FM.

**TABLE 4:** Student’s *t*-test for independent samples comparing groups established by balanced diet, regular exercise, smoking and multi-medication in clinical variables (QoL and severity of FM).

Lifestyle variables		Balanced diet (*n* = 125)				Regular exercise (*n* = 127)			
Clinical variables		YES	NO	t	*P*	YES	NO	t	*P*
QoL	SF-36 mean	35.32 (13.56)	13.81 (2.40)	0.434	0.665	36.93 (13.53)	30.60 (12.46)	2.490	**0.014**
	Physical function	42.06 (19.21)	42.57 (21.76)	–0.126	0.900	45.85 (19.59)	33.20 (17.37)	3.469	**0.001**
	Physical role	27.78 (19.50)	28.59 (23.59)	–0.194	0.846	30.68 (20.13)	22.43 (20.26)	2.125	**0.036**
	Body pain	22.84 (15.47)	20.54 (17.60)	0.707	0.481	24.54 (15.23)	16.71 (16.33)	2.612	**0.010**
	General health	30.23 (16.89)	28.21 (16.28)	0.593	0.554	30.09 (16.95)	28.82 (15.86)	0.397	0.692
	Vitality	25.00 (13.09)	23.78 (13.52)	0.452	0.652	26.53 (12.96)	21.02 (13.03)	2.205	**0.029**
	Social function	41.44 (24.29)	36.36 (23.25)	1.041	0.300	44.31 (23.74)	29.16 (21.71)	3.403	**0.001**
	Mental health	39.86 (14.59)	38.90 (15.27)	0.320	0.749	40.31 (14.87)	38.35 (14.20)	0.694	0.489
	Emotional role	53.63 (28.36)	54.04 (28.26)	–0.072	0.942	53.40 (28.04)	55.12 (28.96)	–0.315	0.753
FM severity	FSQ	21.07 (5.22)	21.09 (4.35)	–0.016	0.987	20.60 (4.56)	22.12 (5.65)	–1.619	0.108

**Lifestyle variables**		**Smoking (*n* = 125)**				**Multi-medication (*n* = 127)**			
**Clinical variables**		**YES**	**NO**	**t**	***P***	**YES**	**NO**	**t**	***P***

QoL	SF-36 mean	29.56 (12.47)	36.79 (13.42)	–2.579	**0.011**	30.64 (12.63)	37.82 (13.34)	–3.024	**0.003**
	Physical function	37.24 (18.83)	43.43 (20.09)	–1.476	0.143	37.30 (18.41)	45.00 (20.13)	–2.177	**0.031**
	Physical role	23.27 (17.66)	29.68 (21.28)	–1.475	0.143	25.12 (19.82)	30.11 (20.74)	–1.347	0.180
	Body pain	17.79 (15.70)	23.68 (15.83)	–1.760	0.081	20.12 (15.49)	23.45 (16.18)	–1.154	0.251
	General health	27.37 (15.06)	30.55 (17.11)	0.763	0.370	28.08 (17.00)	30.72 (16.31)	–0.873	0.385
	Vitality	21.72 (9.47)	25.88 (14.04)	–1.494	0.138	24.00 (12.33)	25.38 (13.75)	–0.579	0.564
	Social function	27.15 (20.61)	43.48 (24.05)	–3.306	**0.001**	32.00 (21.30)	44.64 (24.62)	–2.978	**0.003**
	Mental health	36.00 (14.30)	41.04 (14.55)	–1.641	0.103	36.40 (13.55)	41.87 (15.00)	–2.084	**0.039**
	Emotional role	45.97 (31.46)	56.77 (26.74)	–1.826	0.070	42.50 (26.31)	61.36 (27.06)	–3.879	**0.000**
FM severity	FSQ	22.51 (4.81)	20.74 (4.95)	1.703	0.091	21.58 (4.96)	20.75 (4.96)	0.915	0.365

FSQ, Fibromyalgia Survey Questionnaire; QoL, quality of life. Significant differences (p < 0.05) are marked in bold.

## Discussion

The main objective of this research was to determine which symptoms of fibromyalgia (FM) or lifestyle characteristics predict worse quality of life (QoL) in patients suffering from this disease. The results showed that the most important variable that determine QoL is mood (anxiety/depression scores assessed by the HADS). Also, sleep dysfunction, self-reported pain intensity, and lifestyle/medication variables are associated to QoL, though to a lesser extent.

Firstly, the SF-36 scores indicated a very poor QoL in the patients, as compared to the normative data for the general population. The means obtained in the different SF-36 subscales variables are far below those obtained for the Spanish population (López-García et al., 2003), and close to those obtained by Aparicio et al. (2012) in a sample of patients with FM. These results also agreed with Galvez-Sánchez et al. (2020), who stated that people with FM show markedly lower scores on the SF-36 questionnaire than healthy individuals.

The main role of mood on QoL is consistent with previous research on FM. Better mental health has been related to higher QoL in women with FM (Del Río et al. (2014), while an increase in anxiety resulted in a reduction in QoL (Sempere-Rubio et al., 2019). Galvez-Sánchez et al. (2020) also observed that the presence of comorbid anxiety and depression in people with FM resulted in lower scores on the SF-36 variables.

The regression analysis also showed that pain intensity was a significant predictor of SF-36 scores. Using a measure of experimental pain, Sempere-Rubio et al. (2019) found that a lower pain threshold in women with FM resulted in a lower QoL score. They underscored that the widespread pain characteristic of this disease affects activities of daily life and participation in society (Clauw et al., 2019). It has been suggested that pain has a high negative impact on QoL by generating an increase in stress and anxiety, interfering in daily activities and work, and reducing the positive mental states of people who suffer from it (Galvez-Sánchez et al., 2020). Our results support this idea since we found that mood has a greater contribution than pain itself to QoL.

Concerning sleep quality, we found that the scores of the Pittsburgh Sleep Quality Index also explained QoL in FM patients. In a review on sleep and FM, Choy (2015) stated that sleep disturbance affects to more than 90% of people with FM, and underscored its correlation with pain severity, worsening physical functioning, mood disorders, and fatigue. Also, Wagner et al. (2012) observed that symptoms of sleep difficulty showed an independent, statistically and clinically significant effect on QoL in people with FM. Galvez-Sánchez et al. (2020) defined QoL as the persons’ assessment of both their health and their level of adaptive functioning in daily living activities, including the physical and psychosocial dimensions. Thus, it is understandable that a poorer quality of sleep, which makes it difficult for the body to recover after activities performed throughout the day, results in poorer performance and greater fatigue, as well as in higher pain perception and lower QoL, as indicated by Choy (2015) and Keskindag and Karaaziz (2017).

Regarding lifestyle/medication patterns, the logistic binary regression analysis showed that multi-medication was the variable which best contributed to poor QoL, although the explained variance was low (8.9%). Polypharmacy is a very frequent strategy in chronic pain, not only for elderly patients (Schneider et al., 2021) but also a common practice for children (Gmuca et al., 2019). In spite of the drug-drug interactions and potential harms of poly-medication (Taylor et al., 2013; Giummarra et al., 2015; Menzies et al., 2017), this continues to be the election therapy for FM (Vincent et al., 2015). Very often, patients with FM use not only analgesics (including opioids) but a combination of benzodiazepines, antidepressants, hypnotics, pregabalin, carbamazepine or gabapentin, among others (Gauntlett-Gilbert et al., 2016; Gisev et al., 2019). Some studies with old adults suggest that multi-medication negatively affects QoL (Zhang et al., 2018; Cheng et al., 2020); nevertheless, as far as we know, this has not been systematically studied in FM. Apart from its impact on QoL, we must take into account the high costs that polypharmacy entails for both the health systems and the individual (Taylor et al., 2013).

In line with this, our results show that tobacco use was also negatively related to QoL. Previous studies have reported similar results. Pamuk et al. (2009) and Lee et al. (2011) found that smoking was associated with some psychological factors such as depression, anxiety, and fatigue in FM patients. Tobacco use has been associated with worse QoL in two studies that surveyed large samples of patients with FM (Ge et al., 2019; Croghan et al., 2021). In this regard, it is important to highlight the research carried out by Zale et al. (2014), who found that patients with chronic pain view smoking as a way of coping with pain and even think that abstinence might interfere with pain management.

Although regular exercise was not included in the logistic binary regression models, we observed statistically significant differences in “physical function”, “physical role” and “body pain” between people who exercised regularly and people who did not. Several studies support that regular exercise reduces the functional impact of FM and leads to a higher QoL (Beltrán-Carrillo et al., 2013; Bote et al., 2013). Nevertheless, as discussed by other authors (Beltrán-Carrillo et al., 2013; Del Río et al., 2014; Merriwether et al., 2018), the relationship between exercise and health status seems to be bidirectional: the lesser the limitation on day-to-day activities produced by the disease, the greater the likelihood the person will engage in regular exercise and vice versa.

We also found significant differences between patients who exercised regularly vs. not in “vitality”. As indicated by Merriwether et al. (2018), engaging in daily physical activity is likely to lead to an improvement in fatigue; or conversely, having a lower level of fatigue may allow the patients to exercise regularly. Finally, with regard to “social function”, we found that physical or emotional health problems interfered less with regular social life of those who exercised regularly, as opposed to those who did not. This would be in line with the results found by Beltrán-Carrillo et al. (2013), who argued that group exercise helped participants in their program to improve their social life.

Altogether, the results underscore the role of self-management to improve QoL in FM and may contribute to empower the patients to manage their disease. Promoting positive mood, doing physical exercise and avoiding multi-medication may have positive effects on the patient’s QoL.

### Limitations of the study

Altogether, the symptoms used to predict QoL (SF-36) scores explained 68% of the variance, so it seems necessary to include additional variables to improve knowledge of the key factors which determine QoL in women with FM. For instance, it would be interesting to evaluate the effect of other concomitant psychopathologies or comorbidities. Moreover, we only used dichotomic variables to assess lifestyle influences including items in the clinical interview (i.e., “Do you consider that you are following a balanced diet?”, “Do you do sports?”, “Do you smoke?”). It would be appropriate to deepen on the effect of diet, physical exercise and smoking habits using more appropriate assessment tools, as validated questionnaires or *ad hoc* scales covering more aspects related to these lifestyle variables. Another limitation is that the sensibility and reliability of NRS as a measure of clinical pain have been questioned. We selected the NRS to generalize our results, as it is one of the most widely used measures of clinical pain. However, several studies have highlighted that it may not be the most suitable measure for the study of chronic pain syndromes (DeLoach et al., 1998; de Williams et al., 2000; Chiarotto et al., 2018), and suggest that it could be complemented by multidimensional measures of pain (such as the McGill Pain Questionnaire). Moreover, the definition of multi-medication is currently arbitrary (some studies consider multi-medication the use of two or more drugs -regardless of the type of drug-; others, define it as the use of more than two types of drugs). Thus, there is much heterogeneity among studies that makes it difficult to compare results and draw solid conclusions about medication interactions and side effects on patients with FM. Although our study did not take into account drug doses, we provide preliminary results on the effect of a multi-medication pattern on patients’ QoL, so there is a need to expand knowledge in this field. Finally, it is necessary to point out as a limitation that the sample used in this study was made up of women. We made this decision considering that the percentage of women diagnosed with FM is much higher than the percentage of men (Kueny et al., 2021). However, our results may not be generalizable to the male population diagnosed with FM.

## Conclusion

The results obtained indicate that anxiety and depression were the variables that have more negative influence on the QoL of patients with FM, followed by pain intensity and sleep quality. Considering that FM is a highly disabling disease with a great impact on the lives of people who suffer from it, increasing their QoL is just as important as improving their medical status (Tander et al., 2008). Thus, our data suggest that improving mental health should be a central goal in the treatment of FM. Furthermore, in relation to multi-medication, we observed that it was associated to reduced QoL. Given its high economic costs for the patients and healthcare systems (Taylor et al., 2013) and the associated side effects, this common therapeutic practice for FM should be revised.

Altogether these findings reflect the importance of individualizing treatments and improving self-management practices in this syndrome. It is crucial to progress on the knowledge of the factors that improve the QoL of patients with FM and, above all, on how to modify them, in order to ensure that the advances achieved reach their daily lives. Taking into account our results, we believe that it is particularly relevant to focus on self-management and on how patients themselves can control those variables to improve their QoL.

## Data availability statement

The raw data supporting the conclusions of this article will be made available by the authors, upon request.

## Ethics statement

The study involving human participants was reviewed and approved by the Galician Research Ethics Committee (code: 2014/488) Lugo (Galicia, NW, Spain). The patients provided their written informed consent to participate in this study.

## Author contributions

FF-F participated in data analysis, interpretation of the results, and writing of the manuscript. NS-V participated in data collection and analysis, and supervision of writing. MC participated in the design of the study, supervision of data analysis and results, and review of the entire manuscript. All authors contributed to the article and approved the submitted version.
